# The Modern Western Diet Rich in Advanced Glycation End-Products (AGEs): An Overview of Its Impact on Obesity and Early Progression of Renal Pathology

**DOI:** 10.3390/nu11081748

**Published:** 2019-07-30

**Authors:** Arianna Bettiga, Francesco Fiorio, Federico Di Marco, Francesco Trevisani, Annalisa Romani, Esteban Porrini, Andrea Salonia, Francesco Montorsi, Riccardo Vago

**Affiliations:** 1 Urological Research Institute, Division of Experimental Oncology, IRCCS San Raffaele Scientific Institute, 20132 Milano, Italy; 2 Università Vita-Salute San Raffaele, 20132 Milano, Italy; 3 PHYTOLAB (Pharmaceutical, Cosmetic, Food supplement Technology and Analysis)-DiSIA, University of Florence, 50019 Sesto Fiorentino (Florence), Italy; 4 Universidad de La Laguna, Dep de Medicina Interna, ITB, Instituto de Tecnologías Biomédicas, 38320 La Laguna, Spain

**Keywords:** advanced glycation end-products (AGEs), diet, renal disease, obesity, carboximethyl-lysine (CML)

## Abstract

Advanced glycation end-products (AGEs) are an assorted group of molecules formed through covalent bonds between a reduced sugar and a free amino group of proteins, lipids, and nucleic acids. Glycation alters their structure and function, leading to impaired cell function. They can be originated by physiological processes, when not counterbalanced by detoxification mechanisms, or derive from exogenous sources such as food, cigarette smoke, and air pollution. Their accumulation increases inflammation and oxidative stress through the activation of various mechanisms mainly triggered by binding to their receptors (RAGE). So far, the pathogenic role of AGEs has been evidenced in inflammatory and chronic diseases such as chronic kidney disease, cardiovascular disease, and diabetic nephropathy. This review focuses on the AGE-induced kidney damage, by describing the molecular players involved and investigating its link to the excess of body weight and visceral fat, hallmarks of obesity. Research regarding interventions to reduce AGE accumulation has been of great interest and a nutraceutical approach that would help fighting chronic diseases could be a very useful tool for patients’ everyday lives.

## 1. Introduction

Advanced glycation end-products (AGEs), also known as glycotoxins, are a heterogenous group of molecules characterized by the irreversible covalent bond between a reduced sugar and a free amino group present in proteins, lipids, and nucleic acids [1]. They have long been known since the French physician and chemist Louis Camille Maillard discovered the reaction associated with browning during cooking and storing foods in the early 1900s, but our knowledge about AGEs and their function has considerably increased only in the last 20 years. They are formed physiologically as part of normal metabolism but, when they accumulate at high levels in tissues, they can become dangerous for the organism [2], due to the ability to crosslink proteins and disrupt their structure and thus functions [1]. An exogenous source of AGEs is represented by food and modern western dietary regimens including an elevated consumption of highly processed food and sugar, which are rich in AGEs. Highly oxidant compounds are implicated in the pathogenesis of several inflammatory and chronic diseases. Incorrect lifestyle, including unbalanced diet, and smoking, as well as physical inactivity increase the incidence of metabolic diseases.

This review describes the main features of AGEs and the mechanisms by which they cause pathological alteration in the organism with a special focus on the kidney function. It also illustrates the beneficial effects of dietary AGEs restriction as it occurs in the Mediterranean diet.

## 2. Advanced Glycation End-Products Origin, Structure, and Main Features

**Figure 1:**
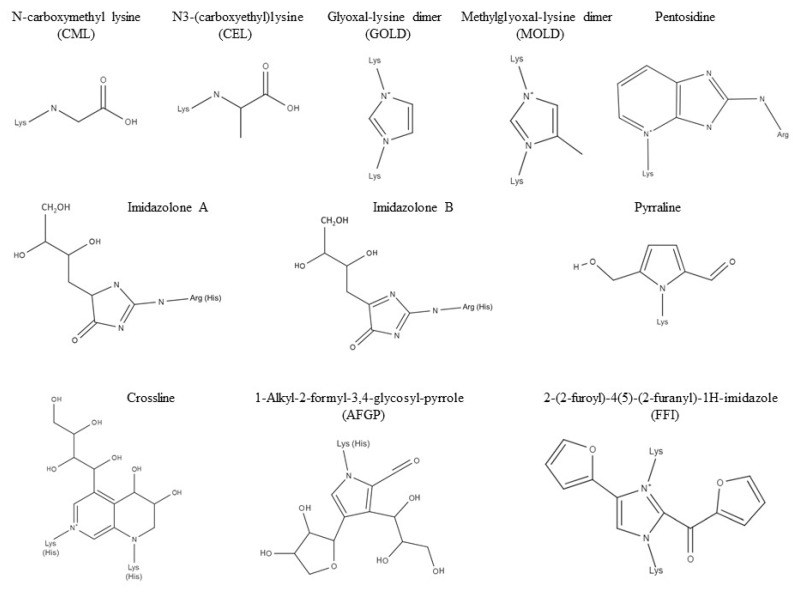
Chemical structures of biologically relevant advanced glycation end-products (AGEs). Lysine (Lys), arginine (Arg), and histidine (His) involved in the crosslinking are shown.

The AGEs family includes a variety of molecules, each with its own structure and characteristics (Figure 1). The majority of reports known to date have all been focusing on the most studied among these molecules, such as carboximethyl-lysine (CML), one of the major AGEs found in vivo and known reactive precursors of the AGE-albumin adduct. Endogenous AGEs are a result of numerous chemical reactions that occur physiologically in the organism. They arise from oxidative and non-oxidative pathways that involve sugars and a variety of heterogeneous reactive precursors such as glyoxal (GO), methylglyoxal (MG), and 3-deoxyglucosone (3-DG). 

In general terms, all the chemical reactions that lead to AGEs can be defined as condensations between nucleophilic and electrophilic reactants [3]. A second way in which AGEs are formed is through a reaction called non-enzymatic glycation, otherwise known as Maillard or “browning” reaction. This reaction involves, as previously stated, the covalent bonding of reducing sugars with free amino groups, either at the protein’s N-terminus and/or at the ϵ-amino groups on lysine’s or arginine’s side chains [1]. In pathological conditions, these reactions generate an excessive amount of AGEs and, if they are not degraded and balanced out by the detoxification mechanisms, this can lead to the activation of the inflammatory response. Exogenous AGEs, on the other hand, originate from the consumption of foods and beverages rich in adducts, as well as the inhalation of combustion products, such as car exhaust or tobacco smoking [4]. Every AGE molecule has its own characteristics and structure, which depend on: (1) The exogenous or endogenous origin, (2) the precursor from which they originate, and (3) whether they are in a free or protein-bound form. Therefore, every AGE adduct has its own metabolic fate, as well as their own potential pathogenic effect. It is important to emphasize that the structures of exogenous AGEs formed during food processing are usually more heterogeneous and complex compared to the ones formed physiologically [5]. However, once the molecule enters the circulation, it becomes undistinguishable in terms of both structure and function and it is not possible to trace them back to their exogenous or endogenous origin [6]. Many studies on AGEs have been carried out using well known AGEs, such as CML or MG, but, given the high number of molecules belonging to this family, it could be incautious to generalize these results for all glycotoxins [7]. 

As previously stated, every AGE molecule has its own structure, which determines their ability or inability to cross-link proteins. Some molecules like, for example, pentosidine, have the ability to cross-link proteins through two covalent bonds, while other molecules only bind irreversibly to one protein, and the well-known molecule CML belongs to this category. The group of inter-related reactions between free amino groups and reducing sugars like glucose and fructose and/or reactive carbonyl precursors such as GO and MG is known as Maillard or “browning” reaction (since it occurs during the processing of food, giving it a characteristic brown–yellow color). The first step in the Maillard reaction sees the creation of a bond through a condensation reaction between the reacting carbonyl group of the reducing sugar with the protein’s free amino group creating a reversible and labile Schiff’s base. This Schiff’s base can undergo spontaneous rearrangements and can isomerize at physiological temperature and pH [3]. This eventually leads to the formation of a more stable ketoamine adduct known as Amadori product. This product has various destinies and one of these is the formation of AGEs, a process that may require months to reach its completion [8]. AGEs can be generated through a one-step conversion of Amadori products, as seen for CML, or a multiple-step reaction that can lead to more complex AGEs like pentosidine or glyoxal-lysine dimer [1]. Amadori products and Schiff’s bases can also generate reactive dicarbonyl precursors such as GO, MG, and 3-DG through reactions called oxidative-fission [9], retro-aldol fragmentation [10], or through a free-radical based mechanism called Namiki Pathway [11]. Dicarbonyls can combine with proteins undergoing a series of reactions, one of which is called Strecker’s Degradation [12]. 

Since the targets of glycation are free amino groups, potentially any protein can be modified, which means that every tissue could be subjected to glycotoxin accumulation [13]. Virtually, any protein can be a target, so, it becomes obvious that the toxic effects of AGEs and their accumulation go hand in hand with the half-life of proteins. Long-lived proteins are, therefore, the most important to study when the subject is AGE accumulation. When talking about proteins with a slow turnover rate, attention should turn to collagen and other Extracellular Matrix components such as laminin and elastin, to the predominant eye lens protein α-crystallin [14], cartilage [15], and also to some plasma proteins such as hemoglobin [1]. However, even though long-lived proteins are more susceptible to glycation, those with a shorter half-life can also be important when dealing with inflammatory effects of AGEs, and the perfect example for this is glycated albumin, which is known to be involved in inflammatory processes leading to renal damage [16,17,18,19]. 

## 3. Receptors for Advanced Glycation End-Products

The discovery of AGEs brought researchers in the past to look for a cellular receptor that could bind them and could, therefore, give rise to the inflammatory response responsible for the pathological effects that were associated to AGEs. The receptor was first described in 1992 and it was termed receptors for advanced glycation end-products (RAGE), given its ability to bind in high affinity AGE molecules. RAGE is the best characterized receptor; however, it is not the only one which is able to recognize and bind AGE-modified proteins. After its discovery, other receptors were identified, and, among these, AGE Receptors 1, 2, and 3 (AGER-1, 2, 3) can be found, as well as the macrophage scavenger receptor CD36 [13]. Evidence suggests that, while RAGE is involved in inflammatory pathways and AGER-2 seems to participate in the early stages of the AGE-activation, AGER 1 and 3 and CD36 receptors are responsible, among other mechanisms, for the detoxification pathways [1,7,20,21,22]. RAGE is a multiligand receptor that belongs to the immunoglobulin superfamily whose gene is located between the major histocompatibility complex 2 and 3 genes on chromosome 6 [23,24]. It is expressed in low quantities in a large variety of cells like macrophages, endothelial cells, neurons, and tubular and glomerular epithelial cells in the kidney [1], while they are expressed constitutively in large quantities in the lungs [25]. Its ligand-binding domain recognizes a large variety of molecules, among which S100 proteins, high mobility group box-1 protein (HMGB1), amyloid β and macrophage 1 antigen complex (Mac-1) and, obviously, AGEs, are the most important [26]. The role of AGEs in inflammatory responses is, therefore, exerted through the activation of this receptor, whose crucial role in innate immunity has been highlighted through RAGE knockout mice models [27]. This study demonstrated that RAGE deficient mice had impaired recruitment of immune cells as well as a markedly lower inflammatory response. RAGE is a 35kDa transmembrane protein of 394 amino acids in length, 19 of which form a single transmembrane domain and 43 constitute a C-Terminal intracellular tail that takes part in the communication with the mediators of the transduction pathways [28]. Other than the membrane bound form, there also are other forms of the RAGE receptor that originate from alternative splicing: sRAGE, which is the soluble truncated form of the receptor that lacks the transmembrane and intracellular domains, and DN-RAGE, which maintains the transmembrane domain but lacks the cytosolic tail in charge of signal transduction [13]. They are thought to suppress the activation of RAGE, functioning either as a decoy for surplus ligands in the extracellular space [29], or as a membrane-bound modulator of the transduction signal in the case of DN-RAGE [26]. The extracellular N-Terminal consists of three separate domains: One V-type domain and two separate C-type domains called C1 and C2 [26]. To date, ligands have been found to bind mainly to the V or to the VC1 tandem domains, while only one study suggests a possibility in binding to the C2 domain [30]. This is explained by the difference in composition of the VC1 domain in respect to the C2 domain: VC1 is mainly composed of positively charged basic residues, while C2 is mainly acidic. This difference in structure outlines a clear subdivision in separate subdomains, which is further reflected by their different ability in ligand binding [26]. All the ligands mentioned earlier may appear completely different from one another, but, in reality, they share common characteristics that could explain why RAGE acts as a non-specific pattern-recognition receptor that may function as an environmental sensor. First of all, they all are negatively charged proteins, which makes it easy for the basic VC1 domain to bind them; second, they all have the tendency to oligomerize and form multimeric proteins [26], which brings us to the next important point, which is how receptors are placed on the cell membrane. In vitro data suggests that RAGE forms multimers on the membrane, which could be formed by four or more receptors linked together through their VC1 domains. This process is called preassembly and it is shared with other receptors of the immune response like TNF-α receptor and interleukin receptors [26]. Preassembly seems to be driven by the multimeric ligands, which bring RAGE clusters to increased oligomerization and result in a stronger transduction signal. Once the ligand binds to RAGE, there is the activation of the so-called AGE-RAGE axis, a series of intracellular cascade events that promote inflammation and tissue damage also through a positive feedback loop that upregulates the expression of the RAGE receptor and accentuates the rise of chronic disease with time [1].

## 4. AGEs and Kidney Disease

Several studies demonstrated that AGE increase in tissues and organs seems to be related to ageing and different pathological conditions, such as diabetes mellitus and uremia. By interacting with their receptors, AGEs are able to promote specific cellular responses, like the release of pro-inflammatory cytokines and profibrotic mediators [31], as described below. AGEs are normally filtered by the glomeruli of kidneys and then reabsorbed by the proximal convoluted tubular district throughout a tricky and changeable clearance mechanism. When AGE blood levels increase, this molecular complex accumulates in the glomerular compartment, promoting an augmented production of type IV collagen and laminin in the extracellular matrix, leading to a pathological process. At the same time, AGEs provoke premature cell senesce and inflammation in the proximal tubule [32]. The kidney plays a pivotal role in the complex system of AGE formation and functions, because the activation of the renin-angiotensin axis can indeed contribute to AGE genesis. In addition, renal podocytes and endothelial cells present specific receptor for AGEs, which help to promote pathological processes such as glomerular hypertrophy and inflammation, interstitial fibrosis and tubular atrophy [33]. Furthermore, a reduction of glomerular filtration rate (GFR), both in acute and in chronic kidney diseases, can rise the amount of circulating AGE in the blood, leading to a faster progression of the primitive submitted nephropathy thanks to the profibrotic activity of AGEs. These pathological effects have been well elucidated in diabetic nephropathy (DN) where AGEs are involved in the microvascular complication derived from hyperglycaemia. In the diabetic renal damage, AGEs synergize and reinforce the asset of other pathogenic actors like the protein kinase C-mediated signal transduction, the renin–angiotensin–aldosterone system (RAAS), and the oxidative stress, resulting in a progressive and self-promoting cycle to chronic kidney disease development [34]. Due to this scenario, much effort has been made to discover different pharmacological drugs able to reduce the circulating levels of AGEs, in order to slow down the progression of nephropathy. However, despite several experimental models in animals have clearly demonstrated a possible role of these tools, a real clinical benefit in humans remains controversial [35]. Few studies have highlighted their involvement also in non-diabetic nephropathies characterized by glomerular sclerosis and tubular dysfunctions. In fact, even in non-diabetic patients affected by a reduction of GFR derived from primitive or secondary glomerulopathies, the normal AGEs excretion is decreased, with the consequence of kidney damage exacerbation due to the prolonged exposition of glomerular and tubular cells to AGE pool [36]. For all these pathological mechanisms and processes, AGE/RAGE complexes and check-point remain an interesting, even though cumbersome, promising pharmacological target in clinical nephrology. 

### 4.1. AGE-Induced Inflammation and Oxidative Stress

In the course of the years, AGEs’ involvement with chronic tissue inflammation has been consistently proven. The main ways in which these molecules contribute to pathological inflammatory state is through the activation of the AGE/RAGE axis and through the oxidative stress. 

The AGE/RAGE axis begins with the interaction between an AGE molecule and RAGE, which is a multiligand receptor belonging to the immunoglobulin superfamily. RAGE activation has been proposed to be involved in the development of food allergies and asthma [37,38], but also in carcinogenesis due to a chronic local inflammation originated, for instance, by *Helicobacter pylori* infection [29]. Ligands that interact with RAGE, such as S100 proteins, HMGB1, and macrophage 1 antigen complex (Mac-1), are all proteins that have roles in immunity as chemotactic agents. Signal transduction pathways involve p21ras, MAP kinases, Rho GTPases, Jun N-terminal kinase (JNK), and JAK/STAT, which lead to nuclear factor migration to the nucleus and the transcription of important genes that regulate chemotaxis, cellular activation, and proliferation [39,40,41,42,43,44,45]. NF-κB, NFAT, STAT, AP-1, ERK1/2, and cAMP responsive element binding protein, bind to their specific promoters for the transcription of genes encoding pro-inflammatory cytokines (i.e., IL-1, IL-2, and IL-4), pro-apoptotic proteins (i.e., p53-Bax that initiates the caspase cascade), and surface proteins such as the vascular cell adhesion molecule, involved in migration [1]. RAGE is constitutionally expressed in many tissues at low level, excepted in the lungs where their levels are markedly higher. Hyper-activation of RAGE causes an excessive stimulation of the PI3K-PKB-IKK pathway, which leads to the binding of NF-κB on the RAGE promoter [46]. RAGE expression on the cell surface is thus improved by an auto-amplification loop where the activation of the receptor leads to increased expression, allowing for further activation [1,13,29]. Its expression can increase due to a rise in extracellular AGEs, but also as a consequence of non-AGE ligand increase, such as the pro-inflammatory cytokine TNF-α [47]. It has been shown that RAGE-activated endothelium promotes recruitment of lymphocytes and delays monocyte apoptosis, prolonging the duration of inflammation [48]. In addition to its function of maintaining and initializing inflammatory responses, RAGEs are also essential for the amplification and modulation of immune cells’ function and growth. 

Oxidative stress is an alteration of physiological conditions in which an imbalance results in oxidant species predominating over antioxidant species [49], and it gives rise to important events such as loss of host defenses and inflammation [20]. Oxidation is a key reaction for the formation of Schiff’s bases and Amadori products and this reaction is frequently supported by oxidant species. Reactive oxygen species (ROS) can be generated in normal physiological conditions, however, when detoxification mechanisms such as antioxidant enzymes (i.e., GSH peroxidase or superoxide dismutase), thiols, and ascorbate are lacking, ROS levels rise and induce proteins, lipids, and nucleotides oxidation. Oxidative stress is increased in renal failure as oxidation products are highly present in patients’ plasma and cellular membranes [49]. Oxidized proteins and lipids take part in the glycation and lipoxidation pathways, being involved in the formation of reactive carbonyl species (RCS) such as GO, MG, and 3-DG. The RCS increase is the link between oxidative and carbonyl stress, and this is the reason why these molecules are so important in the generation of endogenous AGEs. The term carbonyl stress is to be intended as the accumulation of RCS in the plasma that may result in a higher rate of AGEs synthesis and can therefore disrupt the normal balance between AGE synthesis and catabolism [50]. Carbonyl stress is furthermore fueled by the introduction of exogenous AGEs, which can be absorbed by the intestine, degraded and added to the pool of overall RCS and AGEs. AGEs themselves, however, can be a cause of oxidative stress through mechanisms that are either RAGE dependent or independent. This is supported by the involvement of RAGE activation in NADPH oxidase hyper-activation, which is followed by an increased rate of ROS production [51]. NADPH oxidase is a membrane-bound enzyme, which is in charge of pumping electrons deriving from NADPH outside the cell, coupling them with oxygen to produce superoxide ions [52]. In vitro assays demonstrated that incubation with AGEs, such as CML-peptides, increased the level of peroxides in a culture of human endothelial cells, but not in a NADPH oxidase deficient murine model. A reciprocity in activation between ROS and AGEs can be clearly noticed, as AGEs cause accumulation of ROS that in turn cause accumulation of AGEs. 

### 4.2. AGE-Induced Kidney Damage

**Figure 2:**
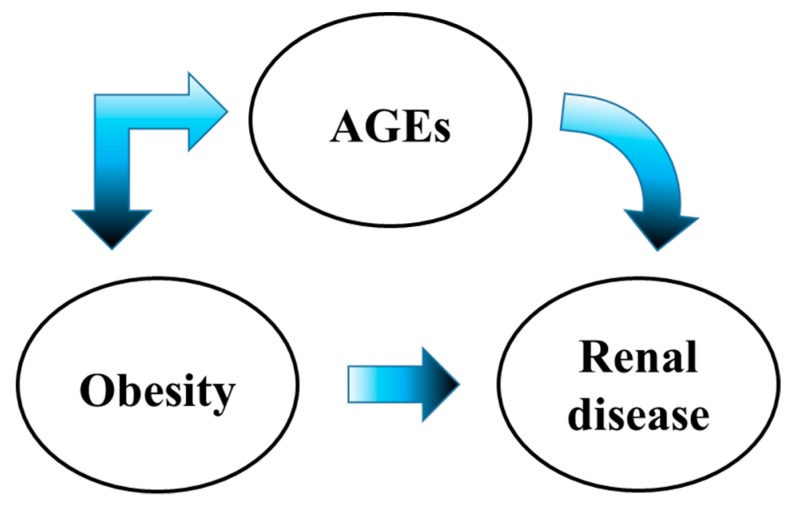
Schematic representation of advanced glycation end-products (AGEs) induced pathogenesis.

AGE-induced activation of inflammatory pathways, their accumulation in tissues and stress affect altogether the structure and function of the kidney. Many of these inflammatory processes leading to kidney damage have been studied in diabetic and non-diabetic animal models for renal disease, as well as in subjects with diabetes, hypertensive nephropathy, obesity-related glomerulopathy, and renal amyloidosis [53]. It is important to consider a possible summation effect of AGEs in patients with diabetes and obesity in the kidney, since *obesity* itself is one of the *most* relevant risk factors for *diabetes* [54] (Figure 2).

ECM metabolism is a delicate balance between deposition and degradation of its components. The main constituent of the ECM is collagen, which is a very long-living protein to which AGEs can bind, and it is probably the most studied when analyzing the products of glycation. RAGE-independent effects on the physiological morphology of the kidney are dependent on the cross-linking ability that some AGE molecules have. When the collagen molecule is glycated, its ability to communicate and bind with other sub-ECM components is impaired, self-assembly of the matrix is compromised and this causes a decreased elasticity of the blood vessels, essential for the filtration in the glomeruli [1]. Glycation of ECM components may, for example, alter the activity of certain metalloproteases and this can lead to mesangial cell expansion and a following thickening of the basal membrane [55], but also to the disruption of tight junctions between the glomerular endothelial cells, which increases glomerular permeability [7]. As for the interaction of ECM components, cell-matrix interaction may also be affected. AGE interaction with type IV collagen and laminin decreases their ability to interact with structures such as proteoglycans and, in the kidney, this can cause an augmented permeabilization of the glomerulus leading to albuminuria [55]. All these altered structural features determine difficult cell growth, loss of cellular adhesion, and alterations in the overall architecture of the epithelium. 

AGEs can generate inflammation through the activation of several cellular pathways that lead to the secretion of pro-inflammatory cytokines, which activate immune system cells and induce tissue damage. The latter triggers repair mechanisms in the damaged area and such chronic inflammation evolves towards fibrosis. In this context, infiltrating macrophages contribute to the alteration of renal ECM structure following their activation due to inflammation processes. In mesangial cells, AGE accumulation stimulates the expression of the monocyte chemoattractant protein-1 (MCP-1) which causes monocyte and macrophage infiltration in the mesangial tissue [56]. This event may facilitate the rise of DN, which correlates with the amount of glycated albumin (Gly-Alb) and glucose during the early stages of the disease. The targeting of MCP-1 and its receptor CCR2 induce an ameliorating effect on streptozoticin treated diabetic mice with renal disease, reducing fibrosis and albuminuria [57]. Since a correlation between AGEs and MCP-1 has been established, a decrease in AGE accumulation in the glomeruli could have a positive effect on the overall health of the renal tissue. Another type of inflammation-derived damage that can be caused by AGEs is the trans-differentiation of renal tubular cells from epithelial to myofibloblasts. This happens through the activation of RAGE that causes the expression of TGF-β and other cytokines that are thought to mediate this differentiation. An in vitro experiment carried out by Oldfield et al. [58] on a rat proximal tubule cell line showed an AGE dose-dependent effect on the secretion of inflammatory cytokines, de novo synthesis of α-actin in smooth muscle and loss of E-cadherin expression. These effects were detectable both in rat cell line and in a human kidney biopsy sample from a type I diabetic patient. 

One of the most characteristic effects of diabetes on the kidney is the loss of the selective glomerular filtration, which results in albuminuria and proteinuria. This selective filtration is based on size and on the presence of podocytes in the glomerulus, which create a barrier that separates the content of blood from what will ultimately become urine. Analysis of renal biopsies revealed that AGEs were diffusely present in the kidneys belonging to DN patients and a positive feedback-loop involving RAGE upregulation was observed also in podocytes [1]. Activation of the NF-κB pathway, together with RAGE amplification and cytokine expression, causes the transcription of the zinc finger E-box binding homeobox 2 (ZEB2) gene [59]. ZEB2 is a transcription factor that can lead to podocyte injury in two ways: First, it suppresses the E-cadherin expression that leads to loss of adhesion due to the expression of N-cadherin. The epithelial-to-mesenchymal transition which occurs causes the detachment from the basement membrane and loss of podocyte count in glomeruli. Second, it causes an alteration of the slit diaphragm composition, which alters function and permeability, by inhibition of P-cadherin and nephrin. The loss of the cadherin proteins and nephrin causes a reduced podocyte count and, consequently, altered glomerular function. The inhibition of NF-κB prevents the activation of the ZEB2 promoter and the ZEB2 knockout blocks the CML-mediated podocyte injury [59]. Taken together, the effects of ZEB2 on podocytes can lead to glomerulosclerosis and thickening of the Bowman’s capsule [7]. A loss of podocytes can also be induced by the AGE-mediated interference of p27(Kip1) with the cell cycle or by the promotion of apoptosis by activation of RAGE-induced FOXO4, a transcription factor involved in the oxidative stress and cell cycle regulation [7]. The Wnt/β-catenin pathway participates in podocyte injury by inducing the expression through NF-κB of several Wnt ligands, which activate β-catenin and induce glomerular filtration impairment and proteinuria [60]. Eucalyptol has already been observed to have anti-inflammatory, anti-viral, and anti-fungal properties, and has already been proven to be effective against diabetes-associated renal tubular epithelial disjunction and tubulointerstitial fibrosis. Eucalyptol was able to ameliorate podocyte injury associated with a marked increase in AGEs by reducing their circulation in the blood stream, by increasing their excretion through urine, by enhancing slit diaphragm proteins’ expression, such as nephrin and podocine, and by interfering with the RAGE-Erk-C-Myc signaling pathway [61]. Mesangiopathy is another result of AGE-mediated damage of the renal tissue. Mesangial cells can also be targeted by AGEs, which, in a similar way to podocytes, alter their cell cycle, inhibit their proliferation, and promote apoptosis and hypertrophy [7]. Through the AGE–RAGE interaction, inflammatory markers and fibrinogenic mediators are increased because of the activation of NF-κB, MAPK, and PKC pathways, resulting in an altered communication and function of mesangial cells with the surrounding cells, and in chronic inflammation and fibrosis of the tissue. The function of the mesangium is of vital importance for the kidney since it is involved in the maintenance of physiological glomerular and capillary tufts; AGE-derived damage to the mesangium results in hyperfiltration [1]. 

Inflammation and fibrosis play a key role in the damaged kidney, but the RAAS is also involved in this process. Aldosterone regulates blood volume and arterial pressure. Renin is a proteolytic enzyme produced in the kidney that causes the conversion of angiotensinogen in angiotensin, which is then transformed by the pulmonary angiotensin-converting enzyme into angiotensin II (AII). AII can act as a vasoconstrictor per se or stimulate the release of aldosterone, which favors fluid retention and increases blood pressure [62]. Local RAAS activity is induced during DN: AII’s activity in early stages of DN is augmented and the increase in blood pressure produces the renal hyperfiltration, which characterizes this disease [63]. AII also causes mesangial and tubular hypertrophy through the activation of NF-κB, which may link back to RAGE activation and expression. An enhancement of inflammatory activity in the kidney mediated by AGEs and AII could occur, with the latter inducing the expression of RAGE, which is then rendered available for AGE-binding. The RAAS has been shown to induce MCP-1 and TGF-β, which could promote even further renal fibrosis and ECM thickening [1]. 

### 4.3. Obesity-Induced Kidney Damage

The deleterious effects of obesity on physiological kidney function are widely known and have been the subject of numerous studies. There are a range of wide-spread complications affecting the renal tissue that are directly linked to the excess of body weight and visceral fat, some of them being mere physical effects and some being more complex metabolic pathways that worsen an already critical situation. Over the years, important correlations between Body Mass Index (BMI) and Chronic Kidney Disease (CKD) have been established. BMI has been observed to be linked to a series of complications affecting the kidney, as reported by Kovesdy et al. [64]. Patients with high BMI, but no pre-existing renal pathologies, have higher risk of renal disease, while the progression of CKD is faster in patients with group 2 obesity or higher with an already diagnosed CKD. Furthermore, higher body fat levels have been linked to a 25% higher risk of developing kidney cancers. On the other hand, the weight alone cannot be the only one responsible for all these complications because obesity is also both a cause and a result of abnormal metabolic activity. The abnormal deposition of fat can affect the kidney directly, altering the expression of pro-inflammatory cytokines and influencing normal oxidative balance, promoting oxidative stress. In this context, various studies have shown how the components of the RAAS are contained in the adipose tissue, making visceral fat deposits actively participate in this system [65]. Higher aldosterone concentrations act directly on the renal tissue leading to an overexpression of the aldosterone receptor and a higher salt reabsorption, which then leads to hypertension and proteinuria. Metabolic Syndrome (MS) has been directly associated to obesity in the course of the years [66]. Even though it does not only occur necessarily in obese individuals, a strong association with visceral fat has been established and their complications on the kidney may have severe outcomes. Among these, we can find insulin resistance and activation of inflammatory pathways such as JNK and NF-κB, which are the same ones activated by visceral adiposity in M1 macrophages [67]. Infiltration of these pro-inflammatory macrophages in the perirenal space could create a channel though which pro-inflammatory cytokines (such as TNF-α, IL-1, and IL-6) can reach the renal tissue, causing inflammation and fibrosis on the long run [68] 

One of the most known effects of obesity on renal function is glomerular hyperfiltration, caused by an increase in plasma flow, filtration fraction, and GFR. Recently, the role of altered physical forces in obese individuals has been analyzed in detail [69]. In glomerular hyperfiltration (GH) conditions, due to an increase in plasma flow, tensile and shear stress (which are the mechanical stresses acting on the glomerulus) are also increased causing different morphological effects on glomerular structures. An increase in tensile stress, which is correlated to blood flowing in the capillaries, causes glomerular base membrane (GBM) expansion, resulting in podocyte foot process lengthening and, subsequently, in podocyte and glomerular hypertrophy. Since the only way by which podocytes can adapt to changes in GBM morphology is through this mechanism, an increase in the shear stress acting on these foot processes has deleterious effects on their function, causing their detachment from the GBM and leading to GH. This process is enhanced in the obese and diabetic patients and is a key link between these diseases and the CKD. According to Tsuboi et al. [65], glomerular hypertrophy has been observed as a result of increased body weight and BMI also in patients without renal pathology or injury, but it is still not fully known whether a decrease in body weight can reverse this condition, regaining normal glomerular size, or if it is an irreversible damage that can easily lead to end-stage renal disease [68]. Glomerular hypertrophy and hyperfiltration are complications that can be found in another condition called obesity-related glomerulopathy. Its incidence and prevalence have increased in the past decade together with the incidence and prevalence of obesity [64]. This disease is characterized by glomerulomegaly and focal segmented glomerulosclerosis, which consists in lesions observed especially in hypertrophied glomeruli, in patients with BMI greater than 30 kg/m^2^ [70]. This disease develops because of complicated and intertwined pathways that include inflammation, hemodynamic changes caused by alterations in renal morphology and physiological balance of the RAAS mediators, and abnormal lipid metabolism and deposition. Recent evidence proved that the adiposity-related increase of aldosterone concentrations is not only responsible for hemodynamic changes in the kidney and abnormal sodium reabsorption, but also participates in the activation of pro-inflammatory signals through the Wnt/β-catenin pathway in podocytes, rendering them more susceptible to apoptosis and basal detachment [71]. 

### 4.4. Do AGEs Participate in the Development of Obesity? 

Although conclusive evidences that indicate consequentiality or causality between AGEs and obesity are still lacking, some connections can equally be suggested. A close positive association between AGEs present in the diet and consumed calorie rates has been reported [72], making it difficult to discriminate the single contributions of each of these risk factors. The main link that can be found is between obesity and inflammatory mediators, in fact it was demonstrated that the chronic exposure to a diet with a high content in dietary AGEs promotes chronic inflammation and insulin resistance, which underlie obesity and MS [73,74]. It has been observed that serum AGEs (ϵN-CML, MG) were markedly higher in obese patients with one other MS criteria compared to others without MS criteria, underlining a correlation with high dietary AGEs, but not with high caloric intake. Higher levels of TNFα were also reported in the same subjects, consistent with evidence that high levels of AGEs promote inflammation and insulin resistance [75]. Accordingly, circulating AGEs have been suggested as a biomarker aimed at identifying the transition from benign obesity to MS, detecting obese people at risk for the development of MS, as well as monitoring the efficacy of dietary interventions [76].

Visceral fat is majorly active in the production of pro-inflammatory cytokines, due to the higher presence of activated macrophages in this district compared to subcutaneous fat, as the concentrations of pro-inflammatory cytokines and RAGE expression are [77]. Macrophage activation was proven to be adipocyte-dependent, given the increased expression of TNF-α, MCP-1, and S100β (among other pro-inflammatory cytokines) and AGE-albumin accumulation. Adipose tissue is a potent pro-inflammatory cytokine producer and adipocytes express a variety of receptors for these molecules [78]. Among these cytokines there are both molecules that can bind to RAGE (S100β and HMGB-1) and molecules whose expression is induced by RAGE (MCP-1, IL-6, TGF-β, etc.), indicating the crosstalk taking place in adipose tissue between the various mediators of inflammation. If inflammation in visceral fat is constantly sustained by macrophage recruitment and activation as well as the RAGE-ligand axis, it allows the large expression of pro-inflammatory cytokines to interfere with normal cellular and systemic physiology [79]. For instance, TNFα increases insulin sensitivity and lipolysis in adipocytes; IL-6 can lead to hyper-triglycemia by increasing lipolysis and hepatic triglyceride secretion [78], while the S100β-RAGE axis is known to be an important regulator of the chronic low-grade inflammation in the adipose tissue occurring in obesity [77]. Chronic inflammation is also fueled by the continuous macrophage recruitment through the increased RAGE-dependent MCP-1 expression, which in turn participate in further RAGE activation, thus creating a RAGE/MCP-1 axis [80]. Chronic activation of the RAGE pathways could, furthermore, amplify all the other pathological mechanisms, increasing oxidative stress and leading to the previously mentioned renal complications. Therefore, a direct causality between AGE levels and the progression of obesity has been hypothesized. An increase in dietary and endogenous AGEs could fuel both oxidative stress and the AGE/RAGE axis, which in turn could increase inflammation in an already inflamed tissue, thus enhancing the progression of obesity. This pathogenic mechanism has been well illustrated by Gaens et al. not only by using an in vitro model of human adipocytes and an obese mice model lacking RAGE on a LeptrDb^−/−^ background, but also by testing it on human models [81]. The authors demonstrated that CML accumulation and RAGE expression are higher in the adipose tissue of obese subjects compared to the adipose tissue of lean subjects. This led to the assumption that reduced circulating levels of CML-AGE observed in the obese human model was due to trapping of CML-AGE via RAGE in the adipose tissue, thereby resulting both in lower levels of circulating CML-AGE and in a dysregulated expression of pro- and anti-inflammatory cytokines. In both preadipocytes and adipocytes, CML incubation increased the expression of inflammatory markers, RAGE, plasminogen activator inhibitor 1, and IL-6, whereas the expression of adiponectin was decreased. The evidence of RAGE-mediated trapping of CML-AGE has been illustrated by the inability of the adipose tissue in mice lacking RAGE on a LeptrDb^−/−^ background to incorporate injected fluorescently labeled CML compared with RAGE-expressing mice [81]. In addition, there are other AGEs receptors able to substantially perturb adaptive adipocyte functions. For instance, CD36 was shown to mediate uptake and degradation of AGEs by adipocytes downregulating leptin in these cells [82].

## 5. Dietary Advanced Glycation End-Products

Thermal processing is a common method in the food industry; flavoring and coloring substances formed through Maillard reaction during high temperature treatments are exploited by food companies with the aim of giving desirable effects to some products [12]. For example, some compounds formed appear brown in color and have characteristic "cooked" aromas, such as freshly baked bread or, if the process was more intense, a toasted-like fragrance, like dried fruit, cocoa, or coffee. This phenomenon occurs in all products containing proteins and reducing sugars and it is accelerated by increasing time and temperature of treatment. Furthermore, it is influenced by water activity (maximum reaction rate at 0,4–0,6 AW value), pH (maximum at pH 10), and size of the sugar reactant (ketose sugars are more reactive than aldoses and pentoses more than hexoses). In addition, during thermal processing, not only AGEs but also advanced lipoxidation end-products can be formed [3]. Non-enzymatic glycation, however, can also occur at physiological temperatures and pH and this is linked to food-specific properties. Years of research have helped in identifying classes of foods that are richer in adducts compared to others. Some tables containing the AGE composition of nutrients included in research papers potentially provide the groundwork for understanding the link between AGE-content and food properties [2,83,84]. Even if researchers have identified CML as a good marker of AGE content in foods (since it is included in both glycation and lipoxidation mechanisms), AGEs are so diverse and heterogeneous that one molecule alone cannot fully represent the real AGE content in foods. This is a very important limitation that can hardly be solved, even using other current chromatographic and immunochemical assays [85]. Another marker for AGE content in foods is MG, but, in contrast with other AGEs like CML, which have an estimated 10% absorption rate in the intestine, reactive dicarbonyls appear not to be absorbable. It is believed that MG cannot reach circulation because it reacts with free amino groups present in the intestine and, therefore, does not exert any effect on the serum AGE levels in vivo [86]. Based on tables and database analysis included in research papers, fats, meat, cheese, and nuts (if processed, canned, or toasted at high temperatures) had the highest AGE content, while dairy, grains, fruits, and vegetables the lowest. Within the meat group the CML contents decrease gradually in poultry, pork, fish, eggs, and lamb [2,87]. The reason for this high AGE content in red meats and poultry is probably given by the fact that, when cooked under dry heat, these release high amounts of highly reactive amino-lipids and reducing sugars, like fructose or glucose-6-phosphate, due to the rupture of lean muscle cells. Even if the fat group is the one that contains the most adducts, it is the meat group that could account more for a high AGE intake since fats cannot make a meal by themselves and the quantities ingested are substantially lower. The fat group, however, can increase the contents of other food groups if the cooking method used requires it. The data shows a substantial rise in AGEs in those foods that have been cooked using butter or oil [83,85]. What appears clear from these first statements is that the fat group and meat group, which have a high lipid and protein content, are more prone to having high AGEs. These foods have a high quantity of lysine and arginine residues, which are, together with cysteine, tryptophan and histidine, favorable glycation targets [88]. Modifications of amino acids (including the essential ones) limit their bioavailability and may lower the nutritive value of a food product. An interesting point can rise from the analysis of protein rich foods like beans, which contain relatively high amounts of lysine and arginine, but do not contain high CML levels. An explanation of this could be related to the nature of carbohydrates: those contained in meat or beans are different, as well as the protein composition and the cooking methods used to prepare them. Not all carbohydrates have the same reducing ability and are not all reactive in the same way. Some literature states that fructose is 8–10 times more reactive than glucose when involved in the formation of Maillard reaction products, due to the higher stability of its open chain form, but also due to the fact that it can generate reactive Heyns products (different from Amadori products) and other fructose-mediated adducts [89].

Even if still dependent on the cooking method, plant-based foods (which are an important part of the Mediterranean diet) such as grains, legumes, fruits, and vegetables are carbohydrate-rich foods with low AGE content. This is rather unexpected since AGEs such as CML are formed from carbohydrates. The reason must be sought in the higher quantity of water and in a richer presence of vitamins and antioxidant molecules, which may be able to prevent AGE formation. Particular notice must be given to Vitamin D [22] and polyphenols derived both from medical and food plants since evidence suggests they have antiglycation activity in vitro and have been seen to prevent AGE formation in vivo [90]. In particular, the results show that polyphenols can inhibit the biosynthesis of AGEs through different mechanisms: Antioxidant processes, protein interaction, metal-chelating ability, MG trapping, and/or blocking the RAGEs [91,92]. Even though there is still no conclusive data regarding the effects of polyphenols on the production of AGEs in vivo, there is strong evidence regarding their role to prevent MG production during food storage and processing [91].

On the contrary, carbohydrate rich foods as chips, crackers, and biscuits present particularly high amounts of CML. The reason for this is not due to the nature of the raw materials which compose these snacks, but rather due to the cooking conditions and to the addition of fatty and sugary ingredients such as butter, oil, cheese, eggs, nuts, or creams that enhance the CML content. Even though their AGE content is far below what is present in meats and fatty food items, their abuse could contribute to an overall rise in the physiological levels of adducts in tissues and thus could represent a serious health risk.

Since the modern Western diet is crammed with heat-processed foods that contribute to the intake of AGEs [93], it is important to consider the choice of cooking methods as a strategy to lower the AGE content in foods. A comprehension of the contribution of different food preparation conditions in AGE formation is important to better outline indications that have to be followed in order to obtain the “perfect” low-AGE diet. AGE formation seems to be reduced by heating in high humidity conditions, shorter cooking times, and lower cooking temperatures.

Goldberg et al. point out in their study that the trend of AGE content in the same food item is oven-frying > deep frying > broiling > roasting > boiling, poaching, and steaming, and suggest that microwaving increases AGE content in foods in a comparable way to boiling [83]. Therefore, different cooking methods such as boiling versus frying and grilling are commonly used in intervention studies. Another way to interrupt or slow down AGE formation is to use acidic ingredients such as lemon juice or vinegar, a practice widely used in the Mediterranean diet. Meat that was marinated with these ingredients, in fact, was shown to have half of the dietary AGEs produced by untreated meat [2]. Due to the fact that a reduction of the AGE content in food can be obtained by simply changing certain culinary techniques and appears to be easily achievable, a dietary intervention following precise meal preparation guidelines could be promising for decreasing inflammation and oxidative stress. Both factors are a cause for the progression of renal pathology itself [72,94], for early onset glomerulomegaly, for hemodynamic changes in a hyper-filtering kidney, and for increased proteinuria, which are all clinical hallmarks of obesity-related renal disease. All this is known since the 1990s, when a central role of the kidney in the regulation of the AGE metabolism was hypothesized, classifying these molecules as potential uremic toxins [95].

Several studies using healthy subtotally nephrectomized rats as animal model for chronic kidney disease demonstrated that all animals gained weight more rapidly with consumption of an AGE-enriched diet and that the most important AGE-induced renal damage was glomerular sclerosis and a dose-dependent rise in proteinuria [96,97]. Moreover, subtotally nephrectomized rats tended to have a higher creatinine clearance, probably as a consequence of AGE-induced glomerular hyperfiltration (as suggested by a higher protein-to-DNA ratio in renal cortex) [98]. Comparing the plasma concentrations of AGEs and the sRAGE in relation to markers of oxidative stress, microinflammation and renal function in obese and lean children/adolescents, the hyperfiltration present in obese children/adolescents has been supposed to be a way to enhance the removal of AGE peptides that partially contribute to the lower plasma AGE levels [99]. In line with this, the Mediterranean diet (which is rich in monounsaturated fatty acids, vitamins, antioxidant molecules, and minimally processed natural foods) could be an example of a low-AGE diet able to reduce circulating AGE levels and to prevent chronic inflammation and oxidative stress. Its effects may be not only linked to the previously discussed dysregulations in the expression of pro- and anti-inflammatory cytokines in different tissues, but also to gut microbiota changes. In fact, recent studies in the nutritional field have illustrated that dietary AGE restriction may result in gut microbiota changes in a population of end stage renal disease patients [100]. It is not known whether these changes are positive or negative, since only a variation in diversity was observed, but no conclusions were drawn. However, there is a modulation of the intestinal microbiota as a result of AGE ingestion. It is known that about 10%–30% of total ingested AGE molecules and Amadori products are absorbed in the intestine [101], but what is not yet known is if the rest of the AGEs have another role other that being expelled through feces. AGEs that are not absorbed may modulate the microbiota because some species possess deglycation enzymes allowing the bacteria to degrade AGEs and precursors in order to use the products, carbon or nitrogen, as a source of energy [102]. This study proved that AGEs modify gut microbial composition and that they could also increase colon permeability in rats. Animals fed with a high AGE diet showed a decreased diversity of microbiota with abundance of *Bacteroides* and *Desulfovibrio* with marked proteolytic activity at the expense of saccharolytic species such as *Alloprevotella* and *Ruminococcaceae*. It was also shown that ammonia and branched chained fatty acids were elevated, suggesting that protein fermentation was enhanced in the colon. High levels of ammonia in the gut was shown to interfere with the cells’ metabolism and morphology, explaining the low expression of tight junctions, which resulted in increased permeability. They, furthermore, explain that changes in proteins caused by heat can decrease their ability to be degraded by upper intestinal bacteria, meaning that they would accumulate in the colon, thus favoring the appearance of more proteinaceous bacteria, which would decrease diversity but also increase the production of putrefactive toxic compound like indoxyl sulfate. *Desulfovibiro* abundance would cause an increase in hydrogen sulphate release induced by high AGE western diets and this gas has been proven to be toxic on epithelial intestinal cells, leading to affected integrity and barrier dysfunction through pro-inflammatory effects associated with inflammatory bowel disease and colorectal cancer. This mechanism, added to all the previously described ones, could pose a serious health risk for the individual since increased colon permeability would allow bacterial translocation with subsequent systemic infections and multi organ dysfunction syndrome. Since the change in the microbiota could be caused in different proportions depending on the type of AGE molecule that is most abundant, there is a need for a broad investigation aimed at characterizing the different effects that varying AGEs could potentially have on the gut microbiota [102]. Nevertheless, how AGE molecules interfere with the gut microbiota is still amply debated. Indeed, there is a lack of agreement on what the changes caused by AGEs on the microbiota may be. As an example, in vivo and in vitro studies reported a contraction in *Bacteroides, Lactobacilli*, and *Bifidobacteria* instead of an expansion [103]. Moreover, it is important to underline that different AGE molecules or AGEs-containing foods were used to prove a change in the microbiota, making them poorly comparable. Indeed, diet enriched in bread crust supplementation with glycated fish protein as a model of a high Maillard reaction product was associated with an increased SCFA concentration, but decreased when animals consumed baked chow [103]. There is a need for standardized protocols with which to test changes in the microbiota, thus resolving the comparative issue and the discrepancies of the results collected. This topic is one of high interest lately in many research fields and it would be fascinating to prove a correlation between high dietary AGE intake, changes in microbiota composition, and diversity and how these changes affect health.

## 6. Conclusions

Food is one of the main sources of exogenous AGEs and studies on different dietary regimens are crucial in order to understand AGE intake, even if it is often difficult to carry out since it needs to be evaluated in the long term and good compliance to the diet is required. In addition, a complete database on AGE content in different foods is still lacking. Interest in healthy diets is increasing worldwide as more and more people become health-conscious. If practical recommendations for correct food choices and cooking methods are combined with physical activity, beneficial results to the lay public may be provided and this would be a significant turning point in improving the health of a world that is too often careless with their own wellbeing. Even though the effects of AGEs on kidney disease are well documented, research on the effects of a high AGE diet on obesity and its complications is less acknowledged. While excluding the possibility that AGEs may be a primary cause for obesity by themselves, their involvement in the development and aggravation of the disease cannot be ruled out. Future research will be essential in outlining the role and significance of these molecules in the progression of obesity, and, despite a breakthrough being still far away, in vitro results are very promising, even if they need to convincingly prove their reliability. With this direction in mind, it is possible to support the expectation that diet contributes to maintaining the health of the kidney as well as delaying progression of inflammatory and chronic kidney diseases. To tailor individual approach to the patient’s clinical status and select the most appropriate nutritional education program, a multidisciplinary health care task force should consider multiple aspects. These aspects should draw from a medical, environmental, and personal point of view in order to provide individuals with advice of consistent quality while remaining relatively convenient for and in line with the patient’s tastes.

The relation between dietary AGEs and the gut microbiota is moving its first steps and could be very fascinating if the hypotheses that have been suggested turn out to be accurate. There is an impelling need to develop standardized evaluation methods that can be used to study this particular topic in order to formulate comparable results. This reasoning stands also for what concerns dietary interventions, since both an upper limit of AGE intake and what defines a high or low AGE diet still have to be recognized. 

Most studies describe the effects that AGEs have extracellularly by binding to receptors and by accumulating in the renal tissue, but interesting ideas for further research on the effects of these molecules on this particular district could also come from the possible intracellular formation of AGEs. So far, in vitro intracellular toxic AGE molecules that participate in cytotoxic effect in the cardiac and hepatic tissues have been identified [104,105]. These molecules, generated as intermediates during the abnormal glucose and fructose metabolisms in the presence of excess glyceraldehyde, are named toxic advanced glycation end-products (TAGE). These glyceraldehyde-derived AGEs apparently participate in apoptotic events and are, therefore, linked to cell death and tissue injury in general. TAGE-induced suppression of autophagy in cardiomyocytes has been recognized as a possible reason for the premature cell death and cardiac disfunction that characterizes patients with cardiovascular disease, opening new perspectives for research in numerous other fields, kidney health included.
